# Genomic Evidence Reveals the Extreme Diversity and Wide Distribution of the Arsenic-Related Genes in *Burkholderiales*

**DOI:** 10.1371/journal.pone.0092236

**Published:** 2014-03-14

**Authors:** Xiangyang Li, Linshuang Zhang, Gejiao Wang

**Affiliations:** State Key Laboratory of Agricultural Microbiology, College of Life Sciences and Technology, Huazhong Agricultural University, Wuhan, P. R. China; The University of Hong Kong, Hong Kong

## Abstract

So far, numerous genes have been found to associate with various strategies to resist and transform the toxic metalloid arsenic (here, we denote these genes as “arsenic-related genes”). However, our knowledge of the distribution, redundancies and organization of these genes in bacteria is still limited. In this study, we analyzed the 188 *Burkholderiales* genomes and found that 95% genomes harbored arsenic-related genes, with an average of 6.6 genes per genome. The results indicated: a) compared to a low frequency of distribution for *aio* (arsenite oxidase) (12 strains), *arr* (arsenate respiratory reductase) (1 strain) and *arsM* (arsenite methytransferase)-like genes (4 strains), the *ars* (arsenic resistance system)-like genes were identified in 174 strains including 1,051 genes; b) 2/3 *ars*-like genes were clustered as *ars* operon and displayed a high diversity of gene organizations (68 forms) which may suggest the rapid movement and evolution for *ars-*like genes in bacterial genomes; c) the arsenite efflux system was dominant with ACR3 form rather than ArsB in *Burkholderiales*; d) only a few numbers of *arsM* and *arrAB* are found indicating neither As III biomethylation nor AsV respiration is the primary mechanism in *Burkholderiales* members; (e) the *aio-like* gene is mostly flanked with *ars-like* genes and phosphate transport system, implying the close functional relatedness between arsenic and phosphorus metabolisms. On average, the number of arsenic-related genes per genome of strains isolated from arsenic-rich environments is more than four times higher than the strains from other environments. Compared with human, plant and animal pathogens, the environmental strains possess a larger average number of arsenic-related genes, which indicates that habitat is likely a key driver for bacterial arsenic resistance.

## Introduction

**Figure 1:**
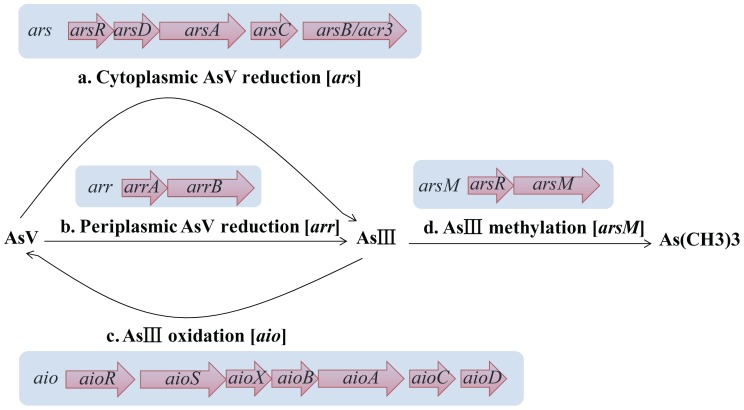
Four major metabolic strategies for arsenic resistance and transformation were found in microbes. a) cytoplasmic AsV reduction by ArsC and As III extrusion by ArsB or ACR3; b) periplasmic AsV reduction under anaerobic conditions by ArrAB; c) As III oxidation by AioAB and AsV extrusion through a phosphate transporter system; d) As III methylation to the gaseous compound As(CH)3 by ArsM. The gene organizations representative of these four processes are shown in the pale blue box, and the corresponding functions of the genes are listed in Table 1.

Arsenic (As) is considered one of the most toxic metalloids widely distributed on earth. Due to anthropogenic pollution and natural transformation, many countries have suffered from arsenic contamination and subsequent poisoning. Arsenic contamination, especially of soil and groundwater, has become a global environmental problem. Microbes play an important role in the global geochemical cycle of arsenic [1], [2]. To adapt to habitats contaminated with arsenic, microbes have developed multiple strategies for resistance to and transformation of arsenic. These strategies have primarily included the following: 1) cytoplasmic/periplasmic AsV reduction and As III extrusion; 2) As III oxidation and AsV extrusion; and 3) As III methylation and volatilization by way of the formation of a gas, also called biomethylation [2]–[6]. These strategies are summarized in Figure 1, and the genes associated with those processes are listed in Table 1.

**Table 1:** Arsenic-related genes involved in bacterial arsenic resistance and transformation.

Family	Gene	Product
*ars*	*arsR*	Arsenic transcriptional regulator
	*arsB*	Arsenic efflux pump
	*arsC*	Arsenate reductase
	*arsH*	Putative flavoprotein
	*acr3*	Arsenic efflux pump ACR3 family
	*arsA*	Arsenite active ATPase
	*arsD*	*ars* operon trans-acting repressor
	*arsO*	Monooxygenase
	*glo*	Glyoxalase/bleomycin resistance protein
	*mfs*	Major facilitator superfamily
*arr*	*arrA*	Respiratory As(V) reductase large subunit
	*arrB*	Respiratory As(V) reductase small subunit
*aio*	*aioA*	Arsenite oxidase large subunit
	*aioB*	Arsenite oxidase small subunit
	*aioX*	Phosphonate-binding periplasmic protein
	*aioS*	Periplasmic sensor, signal transduction, histidine kinase
	*aioR*	Two component, sigma54 specific, transcriptionalregulator, Fis family protein
	*aioC*	Cytochrome c, monoheme
	*aioD*	Molybdenum cofactor biosynthesis protein A
*arsM*	*arsM*	Arsenite S-adenosylmethyltransferase

Note: *ars*, cytoplasmic AsV reduction; *arr*, periplasmic AsV reduction; *aio*, arsenite oxidation; *arsM*, arsenite methylation.

In the past, arsenic-related genes have been reported to be widely distributed in bacterial genomes. The sequences of genes such as *arsC*, *arrA*, *arsB/acr3*, *arsM*, *aioA* and *aioB* displayed significant diversity, as determined through PCR-based approaches [1], [7]–[11] and high-throughput metagenomic approaches [12]–[14]. The PCR-based method is highly dependent on the coverage and specificity of the universal primers used to target the genes of interest. This method can underestimate the abundance of arsenic-related genes if multiple copies of the genes were present in the bacterial genome. As for the high-throughput metagenomic approach, certain false positives would occur due to very small read lengths (approximately 100 bp for an Illumina sequencer and 400–600 bp for a Roche 454 sequencer). Furthermore, this approach could not associate specific genes with the respective strains. Therefore, both approaches lack the complete and reliable information regarding the distribution of arsenic-related genes in individual bacteria. With the rapid development of high-throughput sequencing technology, a large number of microbial genomes have been sequenced in recent years. There is no doubt that genomic sequence of a strain contains nearly all of the information about its arsenic-related genes. Therefore, in this study, we used genomic information to investigate the distribution, abundance and organization of arsenic-related genes in bacteria.

We employed the genome sequences of strains in the *Burkholderiales* order as a case study to assess the evolution of arsenic related genes. We chose this order based on the numerous factors. 1) Strains in this order display phenotypic, metabolic and ecological diversity, which included bacteria from different niches and lifestyles [15]. 2) To date, a large number of genomes have been sequenced in *Burkholderiales*, and approximately 215 genomes are available in the National Center for Biotechnology Information (NCBI) database. These available strains include all five families: *Burkholderiaceae*, *Oxalobacteraceae*, *Alcaligenaceae*, *Comamonadaceae* and *Sutterellaceae*, as well as the unclassified family. 3) Many previously reported arsenic-resistant and arsenite-oxidizing strains belong to this order, and their genome sequences have been determined [16]–[20]. In the present study, we systematically re-annotated the arsenic-related genes based on protein-similarity, and we compared the relationship between the distribution of arsenic-related genes in strains and their habitats. With the results of this new analysis, we discuss the evolution of arsenic-related genes along the phylogeny of the *Burkholderiales* order.

## Materials and Methods

### Genome sequences and annotation

All available genomes of strains belonging to the *Burkholderiales* order were retrieved from bacterial genome database in NCBI, including 91 complete and 124 draft genomes (genomes available as of Jan 21, 2013). Among the 124 draft genomes, some genomes lacked annotation information. Therefore, we annotated these genomes with the RAST high-quality annotation system [21] using Glimmer 3.0 gene prediction software [22], and the annotation results are stored online (rast.nmpdr.org/; account: smark1984; password: 397310). In addition, the draft genomes with contig number greater than 1,000 were excluded from the analysis if their original genomic annotations were unavailable. In total, 188 genomes were used for the analysis presented in this study.

### Phylogenetic analyses

The 16S rRNA gene-based tree was a fast and easy approach to reconstruct the phylogeny of the targeted strains. We first analyzed the phylogeny of these 188 strains using 16S rRNA genes. However, the 16S rRNA gene-based tree of these 188 strains could not clearly distinguish them. Thus, a phylogenetic analysis based on the conservation of proteins shared across the 188 genomes was performed. The conserved proteins of these 188 genomes were identified with blastP, using an “all vs. all” strategy. Based on the blastP analysis (threshold value: e-value = 1-e^10^; coverage > =  70%; identity > =  50%), the 188 genomes contained 10 conserved genes that had exactly one member per genome, and the lengths of each of the genes were nearly identical. Each set of the conserved proteins was aligned by clustalW [23], and all of the sets of the alignments were concatenated into a string of amino acids for each genome. Finally, the concatenated alignment data were used to infer phylogenetic relationships by PhyML with a maximum-likelihood (ML) algorithm [24]. One-thousand bootstrap repetitions were used to estimate tree reliability.

### Arsenic-related gene annotation

Due to the extreme diversity in arsenic-related genes (such as *arsR* and *arsC*) [25], the annotated information of the genomes in NCBI or in the RAST system may include incorrect annotations for numerous genes. For example, some of the arsenic-related genes were annotated with other names. Thus, it is not appropriate to identify these genes simply by the names of their proteins. Therefore, we extracted the arsenic-related gene information according to our re-annotation strategy, as illustrated in Figure S1. First, we built a preliminary-screening database by gathering the arsenic-related sequences from the NCBI protein database. All of the predicted proteomics sets from these 188 genomes were searched against this “self-build arsenic database” using the blastP algorithm, and we used a custom Perl script to parse the blast results with conventional criteria (e-value = 1-e^10^; coverage> =  70%; identity > =  35%) to obtain the candidate genes. The candidate genes were filtered through protein functional classification, Clusters of Orthologous Groups (COG) [26] and ortholog clustering analyses by OrthoMCL, with an inflation value of 1.5 [27]. According to the results that we obtained, the relatively pure arsenic-related genes were divided to two groups (scattered genes and genes clustered together) by a manual analysis. Apparently, the genes clustered together were the actual arsenic-related genes. The scattered genes were searched against the genes that clustered together for further confirmation.

### Heatmap analysis of the distribution of arsenic-related genes

To clearly display the distribution of the arsenic-related genes in these 188 strains, we made a matrix with 188 rows and 21 columns, in which the rows represented the 188 strains and the columns represented an individual arsenic-related gene or *ars-like* cluster in each strain. From top to bottom, the 188 strains were ordered according to the sequence of the strains in the core genes-based phylogenetic tree. This matrix was used to produce a heatmap with a custom script written in the R based language (http://www.r-project.org/).

## Results

### Overall information on the 188 *Burkholderiales* genomes

**Figure 2:**
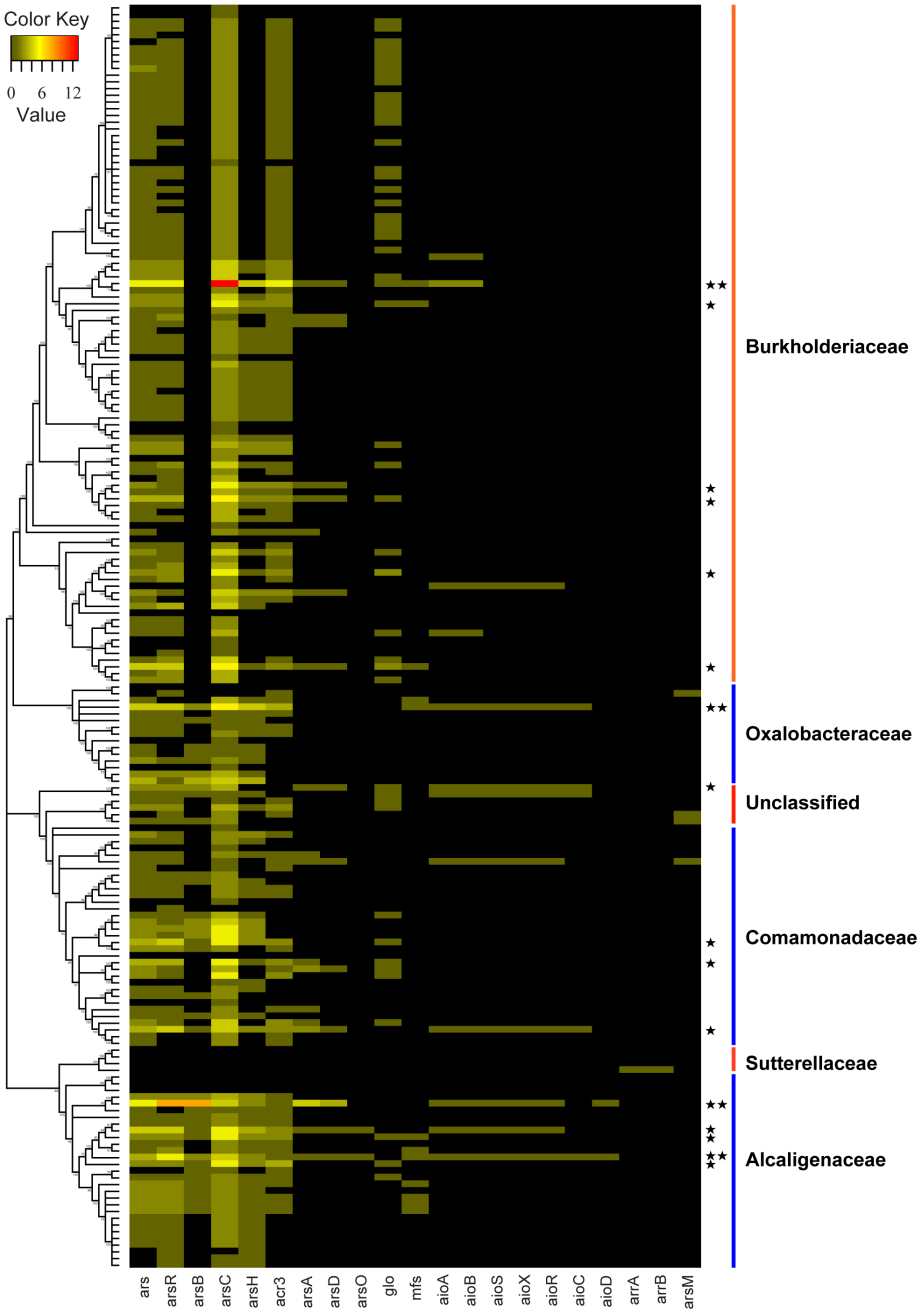
Distribution of arsenic-related genes in 188 *Burkholderiales* genomes. From upstream to downstream in the 10 core genes-based tree, the 188 strains' names and their detailed distribution of the arsenic-related genes is listed in Table S3. The color of the bar indicates the gene numbers. One asterisk and double asterisks represent two times or four times as many as the average number of arsenic-related genes per genome, respectively.

As of Jan. 21^th^, 2013, 215 strains in the *Burkholderiales* order have been sequenced, and most of these strains are involved in pathogenicity and other bio-applications (http://www.ncbi.nlm.nih.gov/genome/?term=Burkholderiales). To associate the distribution of arsenic-related genes with their phylogenetic affiliation, we first tried to determinate the phylogenetic structure among these strains. Our analysis was based on the core genomes of these strains rather than 16S genes because the 16S gene-based phylogenetic tree made it difficult to distinguish the actual relationships (Figure S2). To maintain a suitable size of core genes, 188 genomes were selected for phylogenetic interference and used for the subsequent analysis in this study (Table 2). Based on our analysis, 10 genes were shared among the 188 genomes, and these conserved proteins were used to construct a ML tree. As shown in Figure 2, the core gene-base tree could clearly group the strains into five families and one unclassified family, representing 35 genera and 70 species. The selected 188 strains were distributed among a diversity of ecological sites. According to the isolation sources [15], we could classify these strains into different groups (Table S1). These groups include the following: (i) human host (58 strains, denoted **H** in Table S1), (ii) plant pathogens (14 strains, **P**), (iii) animal host (11 strains, **Z**), (iv) rhizosphere and root nodules (27 strains, **R**), (v) soil (25 strains, **S**), (vi) sediment (7 strains, **D**), (vii) wastewater and sludge (23 strains, **W**), (viii) endosymbionts (3 strains, **E**) and (ix) miscellaneous sources (12 strains, **U**). In addition, the isolation sources of eight strains were unavailable (denoted **NA** in Table S1). Among these strains, *Achromobacter arsenitoxydans* SY8, *Acidovorax* sp. NO1, *Alcaligenes faecalis* subsp. *faecalis* NCIB 8687, *Herminiimonas arsenicoxydans* ULPAs1 and *Thiomonas* sp. 3As were the sequenced arsenite oxidizers isolated from niches contaminated with arsenic, in which, the mechanisms related to arsenic resistance and arsenite oxidation have been widespread investigated [16]–[19], [28]–[33].

**Table 2:** Phylogenetic information on the 188 *Burkholderiales* bacterial genomes.

Family	Genus	Number	Total
*Alcaligenaceae*	*Achromobacter*	6	29
	*Advenella*	1	
	*Alcaligenes*	2	
	*Bordetella*	16	
	*Pusillimonas*	1	
	*Taylorella*	3	
*Burkholderiaceae*	*Burkholderia*	78	101
	*Cupriavidus*	7	
	*Pandoraea*	1	
	*Polynucleobacter*	2	
	*Ralstonia*	13	
*Comamonadaceae*	*Acidovorax*	11	33
	*Alicycliphilus*	3	
	*Comamonas*	4	
	*Delftia*	2	
	*Hydrogenophaga*	1	
	*Hylemonella*	1	
	*Limnohabitans*	1	
	*Polaromonas*	3	
	*Pseudacidovorax*	1	
	*Ramlibacter*	1	
	*Rhodoferax*	1	
	*Variovorax*	3	
	*Verminephrobacter*	1	
*Oxalobacteraceae*	*Collimonas*	1	15
	*Herbaspirillum*	9	
	*Herminiimonas*	1	
	*Janthinobacterium*	1	
	*Oxalobacter*	2	
	Unclassified	1	
*Sutterellaceae*	*Parasutterella*	1	4
	*Sutterella*	3	
Unclassified	*Leptothrix*	1	6
	*Methylibium*	1	
	*Rubrivivax*	2	
	*Thiomonas*	2	

### Overall distribution of arsenic-related genes in *Burkholderiales* genomes

One-hundred and eighty eight genomes were investigated in detail to ascertain the distribution and organization of the arsenic-related genes based on our three-step re-annotation strategy (Figure S1). The number of arsenic-related genes detected in each genome was highly variable, and ranged from zero in the following ten strains [all three *Sutterella* strains (*S. parvirubra* YIT 11816, *S. wadsworthensis* 3_1_45B and *S. wadsworthensis* 2_1_59BFAA), all three *Taylorella* strains (*T. asinigenitalis* MCE3, *T. equigenitalis* ATCC 35865 and *T. equigenitalis* MCE9), *Cupriavidus necator* HPC(L), *Oxalobacter formigenes* HOxBLS, *Polynucleobacter necessarius* subsp. *necessarius* STIR1 and *Verminephrobacter eiseniae* EF01-2] to 35 in *Burkholderia multivorans* ATCC 17616 and 36 in *A. faecalis* subsp. *faecalis* NCIB8687 (Table S3). A total of 1,117 arsenic-related genes were identified in these genomes. Among these genes, 795 genes (71.2%) were grouped into an *ars*/*aio* cluster (at least two arsenic-related genes gather together at position). This result indicates that arsenic-related genes tended to group together. The distribution of arsenic-related genes is presented in Figure 2 and detailed in Table S2. According to the pathways of arsenic-resistance and transformation, there are 1,051 *ars-like* genes, 60 *aio-like* genes, two *arr-like* genes and four *arsM* genes. In our analysis, the *ars-like* genes are the predominant type of arsenic-related gene. In contrast, *arr* and *arsM* were identified only in a few genomes (Figure 2). A set of *arrAB* was only identified in *Parasutterella excrementihominis* YIT 11859, belonging to *Sutterellaceae* family. As for arsenite methylation, *Oxalobacter formigenes* OXCC13 in the *Oxalobacteraceae* family, *Rhodoferax ferrireducens* T118 and two *Rubrivivax*strains (*R. benzoatilyticus* JA2 and *R. gelatinosus* IL144) in the *Comamonadaceae* family were found to contain *arsM* genes. Twelve strains have genes encoding arsenite oxidase, and these strains were located in all of the families except *Sutterellaceae*. In addition, *B. multivorans* ATCC 17616 contained two sets of *aioAB* in its genome. The *aio-like* gene was found in the plasmid of *Ralstonia solanacearum* PSI07. Nearly 95% strains (178 out of 188) harbored arsenic-related genes in their genomes (Figure 2), which indicates that arsenic-related metabolism is widely present in *Burkholderiales* genomes.

The genome size of the 188 strains in *Burkholderiales* varied markedly, from 1.56 Mb (*P. necessarius* subsp. *necessarius* STIR1) to 11.29 (*Burkholderia terrae* BS001) Mb. Inevitably, genomes of a larger size had a greater number of genes. For example, some types of genes that are associated with resistance to antibiotics and toxic compounds, such as multidrug resistance (MDR) efflux pumps, have been reported in greater numbers if the strain has a larger genome [34]. However, unlike MDR efflux pumps, according to our statistical analysis, there was not a positive correlation between genomic size and the number of arsenic-related genes (r = 0.121; p>0.05).

### The *ars* gene is highly abundant and has extreme diversity in its organization

The diversity of arsenic-related genes is reflected by of the *ars-like* genes, which made up 94.1% of the arsenic-related genes and were abundant in 174 strains. Overall, 5.6 *ars-like* genes per genome were observed in *Burkholderiales* strains (Table S2 and Figure 2). As shown in Figure 2, nearly every strain contained several copies of the *arsC* gene in their genomes. The *arsC* gene encodes arsenate reductase and is involved in the transformation of AsV to As III, which is then excreted by the arsenic efflux pump ArsB or ACR3. This mechanism benefits the bacteria itself, though it enhances the toxicity of the surrounding environment. The arsenite efflux pump could be classified into two types, ArsB and ACR3, based on different structures [35]. A total of 205 arsenite efflux pumps were identified in these genomes, including 151 copies of ACR3, which indicates that ACR3 is the primary form of arsenite efflux pump in *Burkholderiales*. Moreover, in the *Burkholderiaceae* family, the arsenite efflux pump was only present as the ACR3 type (Figure 2 and Table S3).

**Figure 3:**
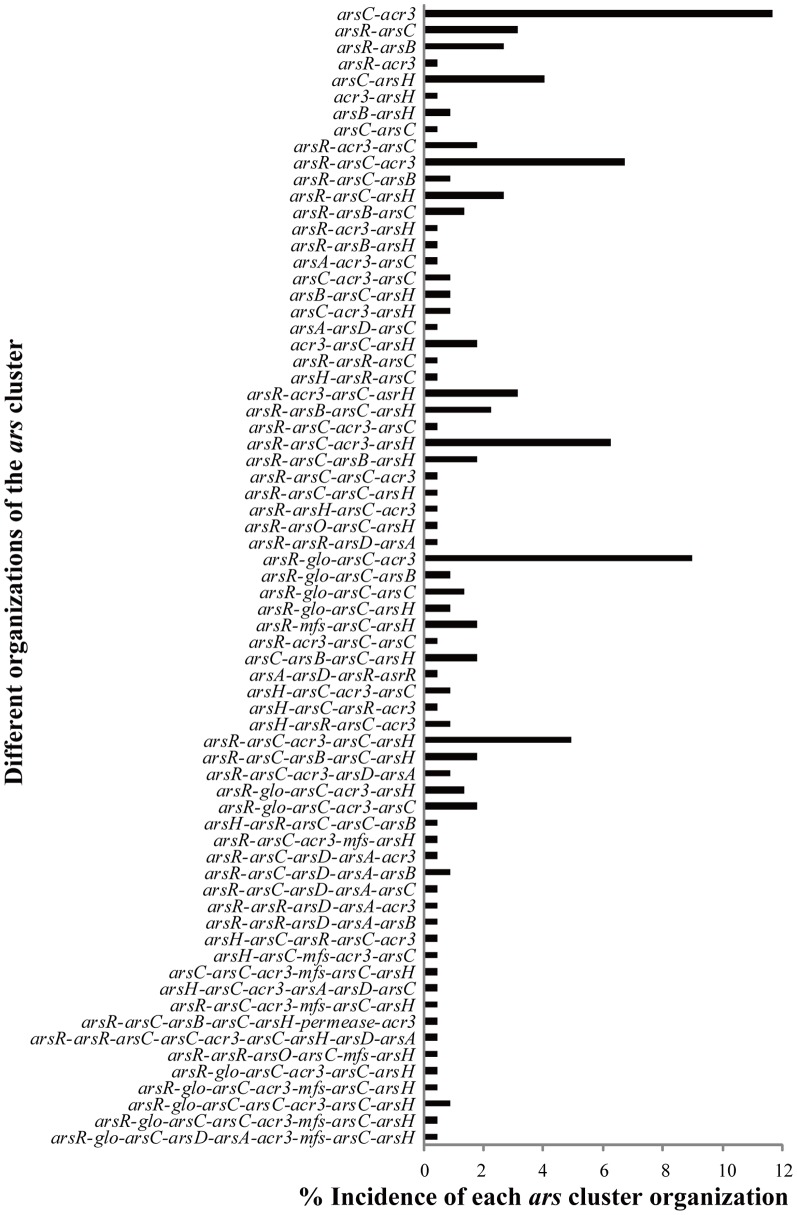
Diversity of organizations of the arsenate-resistance operon (*ars*) cluster in the 161 *Burkholderiales* genomes.

There are a total of 223 *ars* operons identified in 161 strains covering 2/3 *ars*-like genes (Figure 2). As shown in Table S3, 11 strains (*A. arsenitoxydans* SY8, *Achromobacter piechaudii* HLE, *Acidovorax* sp. JS42, *Acidovorax sp.* NO-1, *A. faecalis* subsp. *faecalis* NCIB 8687, *B. multivorans* ATCC 17616, *Burkholderia phytofirmans* PsJN, *Delftia acidovorans* SPH-1, *Herbaspirillum* sp. GW103, *H. arsenicoxydans* ULPAs1 and *Ralstonia pickettii* 12D) contained no less than three sets of *ars* operons in their genomes. According to their organizations, 223 *ars* operons contained 68 different forms (Figure 3).

### Rare distribution of the *arr-like* gene in *Burkholderiales* genomes

Two-gene clusters (*arrA* and *arrB*) are involved in arsenate respiratory reduction, which is found in bacterial and archaea mainly isolated from aquifers and sediments. Of 188 strains, we found that only *P. excrementihominis* YIT 11859 contained one set of *arrAB* genes (Figure 2 and Table S2). The respiratory As(V) reductase large subunit ArrA and small subunit ArrB of *P*. *excrementihominis* YIT 11859 shared 46% and 42% identities, respectively, with those of *Shewanella* sp. ANA-3 [36]. In the *Shewanella* sp. ANA-3 genome, the *arr* cluster was flanked an *ars-like* cluster of *arsD-arsA-arsB-arsC* [36]. However, no *ars-like* genes were identified in *P*. *excrementihominis* YIT 11859.

### Comparison analysis of the *aio* operon and flanking sequences

**Figure 4:**
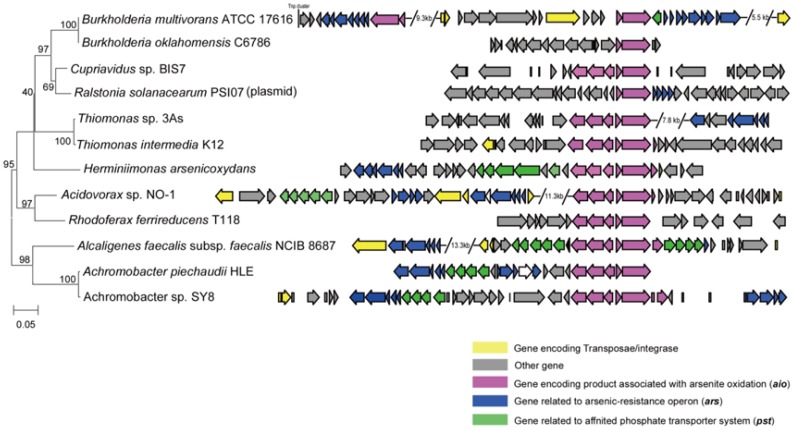
Multiple organizations of the *aio* gene cluster and flanking sequences were detected in arsenite-oxidizing bacteria in *Burkholderiales*.

**Figure 5:**
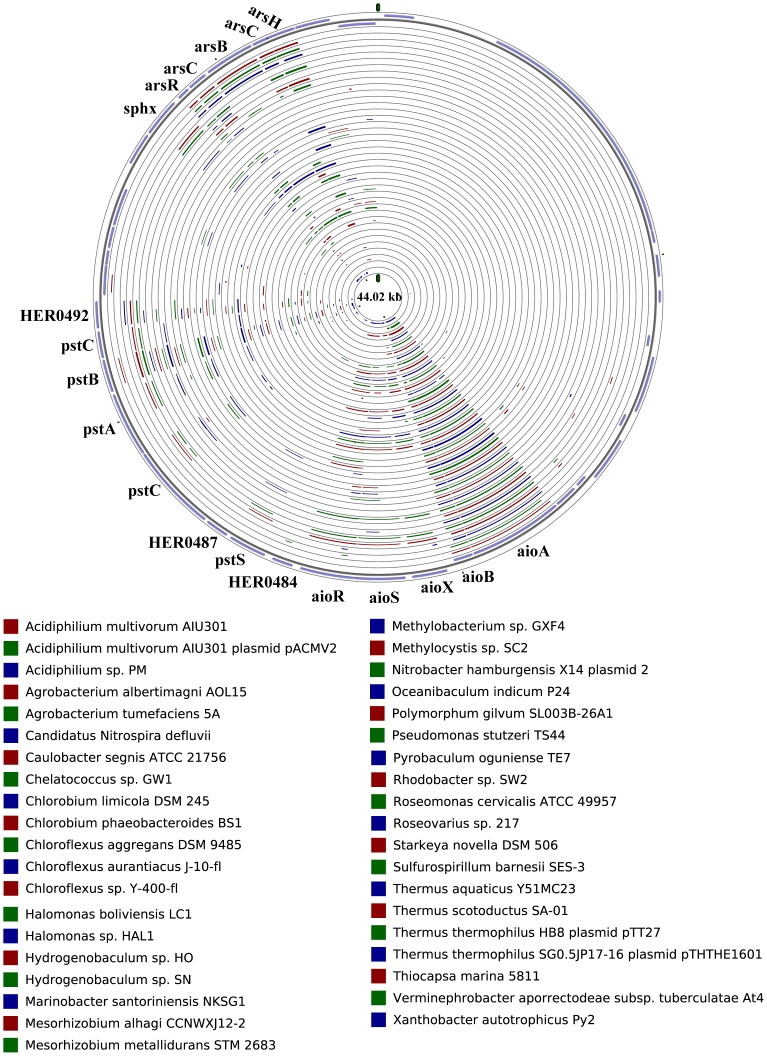
Comparisons of the organization of the *aio* cluster and flanking sequence in 39 arsenite oxidizers genomes. *H. arsenicoxydans* ULPAs1 is used as the reference genome. From outside to inside, first two rings donated ORF encoded from forward/reverse strand of the partial region of the *H. arsenicoxydans* ULPAs1 genome; rings 3 to 41 represent the 39 arsenite oxidizers at this order, which are shown under the cycle (from up to down and left to right).

Bacterial arsenite oxidation transforms the more toxic As III to the less toxic AsV, which is considered an environmental detoxification pathway. Twelve strains were identified that carry *aio* operons in their genomes, among which only *R. solanacearum* PSI07 contained the *aio* operon in its plasmid. The organization of *aio* operons can be roughly grouped into two forms by the presence or absence of the three-component system AioX/AioS/AioR (Figure 4). The *aio* operon is frequently flanked with *ars* operons and genes encoding the high-affinity phosphate transport system *pstSCAB*, as is the case in the other 39 genomes identified in all of the sequenced genomes of bacteria and archaea from the NCBI database (Figure 5). However, comparison of the organization of these *aio* operons revealed a limited synteny of their flanking elements, which may indicate that the *aio* operon was obtained through horizontal gene transfer (HGT).

The *aio* operon appeared to be randomly distributed in four families and the unclassified family of *Burkholderiales*, which is consistent with prediction described above (Figure 2). Although two types of *aioAB* were found throughout bacteria and archaea [37], the small number of strains carrying *aio* operons indicated that the capacity for arsenite oxidation by microbes is a relatively rare compared with that of the *ars* operon resistance system.

### Distribution of *arsM-like* gene in *Burkholderiales* genomes

Microbial methylation of arsenite is mediated by *arsM* and has been found to be widespread in bacteria, archaea and eukarya [38]–[40]. The volatilization of As III in this process is thought to contribute to the global cycle of As. Based on a protein-similarity search, the *arsM* gene was identified in *O. formigenes* OXCC13 (Feature_id, 556269.7. peg.1267), *R. ferrireducens* T118 (Locus_tag, Rfer_1612), *R. benzoatilyticus* JA2 (RBXJA2T_04893) and *R. gelatinosus* IL144 (RGE_20810) (Figure 2 and Table S2). The *arsM* gene was mostly followed by *arsR*, which is believed to control the expression of *arsM*. As for our four *arsM* genes, we found one strain that did not contain *arsR* upstream to *arsM* (*R. ferrireducens* T118), which may suggest that *arsM* is constitutively expressed in *R. ferrireducens* T118.

### Habitat influences the distribution of arsenic-related genes

**Figure 6:**
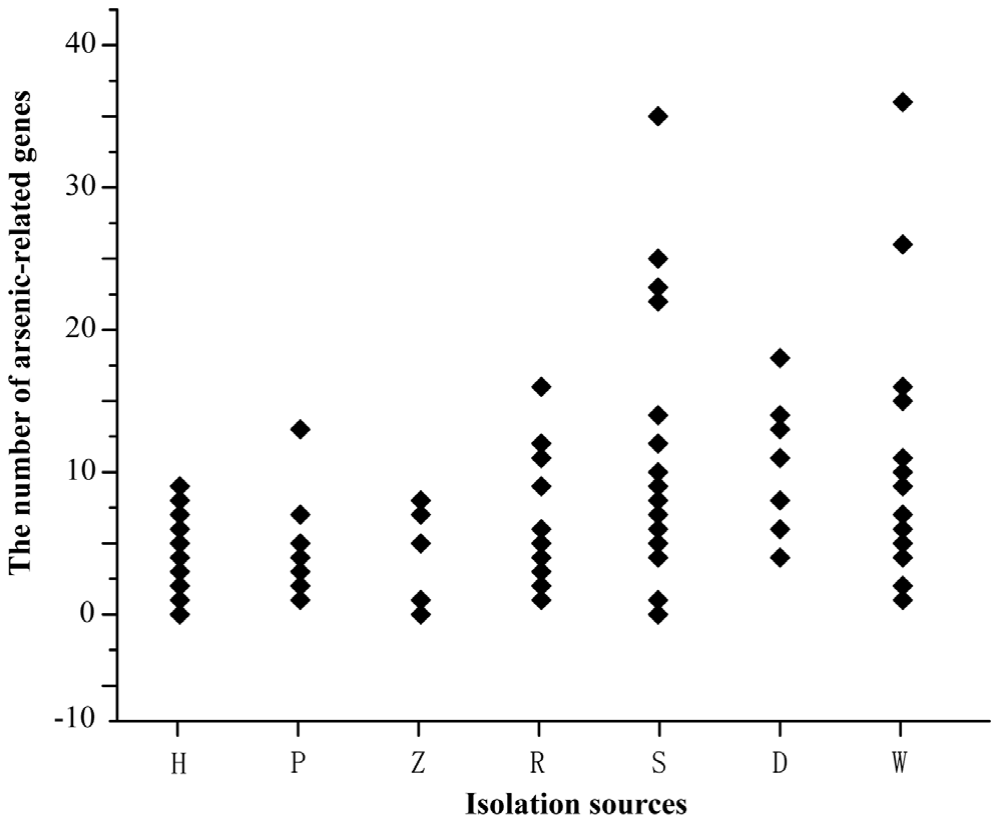
Habitat impacts the distribution of arsenic-related genes in *Burkholderiales*. The scatter distribution of the number of arsenic-related genes per genome grouped by the isolation sources. The isolation sources included human (H), plant (P), animal (Z), rhizosphere or root nodules (R), soil (S), sediment (D) and wastewater or sludge (W) (Table S1).

Compared among the abundance of arsenic-related genes of strains isolated from human, plant, animal, soil, sediment, wastewater or sludge and rhizosphere or root nodule, certain correlations were found: a) the number of the arsenic-related genes of strains isolated from soil (S) and wastewater or sludge (W) are larger than that of strains in the other environments (Figure 6); b) the six strains having more than 20 arsenic-related genes were recovered from S or W, and four of them are from arsenic-rich environment (Figure 6); c) the average number of arsenic-related genes per genome of human, plant and animal pathogens (H, P, Z) was less than that of strains isolated from S, W, sediment (D) and rhizosphere and root nodules (R) (Table S1, Table S3 and Figure 6), and d) the five isolates from the arsenic-rich niches (Table S1) contained more than four times average arsenic-related genes per genome compared to the other strains (25 vs 6 genes, Table S3 and Figure 2).

## Discussion

Previously, many studies have revealed the widespread distribution of arsenic-related genes in bacteria, and arsenic-related genes have been isolated from a large number of bacteria from different niches [1], [4], [8], [9], [11], [13], [41]. In light of these data, it has been assumed that arsenic-related genes were common in all bacteria, but clear evidence has been lacking. To date, numerous bacterial genomes (more than 10,000) have been sequenced. When looking through these genomes, nearly all of the genomes contain some arsenic-related genes despite the strains having been sampled from low-arsenic or arsenic-free habitats. This phenomenon puts us in mind to ensure the feasibility of using mass genomic information to detect the presence of arsenic-related genes in any bacteria. In this study, for the first time, we systematically analyzed the distribution and organization of arsenic-related genes using genome data from strains of *Burkholderiales.* Our studies provided the definitive evidence that nearly all *Burkholderiales* strains contained arsenic-related genes. This conclusion can most likely to be extended to all bacteria, despite the absence of direct evidence in this study. We could speculate that evolutionarily ancient microbes were exposed to “an arsenic surroundings” on ancient earth [42]. To overcome these selective pressures, microbes obtained numerous arsenic-related genes in their genomes for survival. Therefore, the arsenic-related gene may have very early origins, especially the *ars-like* gene. This speculation was supported in part by recent literatures showing that bacterial arsenic resistance and transformation was an acquired trait via HGT, driven by adaptation to habitats containing arsenic [17], [19], [43]–[45]. However, we found that the arsenic-related genes were absent in ten of the 188 examined strains, which suggest that some microbes may lose their arsenic-related genes during adaption to arsenic-free niches. In addition, the number of arsenic-related genes of strains isolated from arsenic-rich environments is much higher than the strains from other environments. Compared with human, plant and animal pathogens, the strains isolated from environmental sources possess a larger number of arsenic-related genes, which suggests that habitat likely plays an important role in influencing the distribution of arsenic-related genes [18], [45], [46].

The *ars-like* genes were highly abundant and displayed an extreme diversity in distribution. The *ars-like* genes were often found in the form of a cluster/operon, but they were also present as a scattered distribution, especially *arsC*. The diversity of organization of the *ars-like* cluster was very significant, and we observed up to 68 forms in 188 *Burkholderiales* strains (Figure 3). Previous research has demonstrated that the three-gene *arsRB(/acr3)C* and five-gene *arsRDAB(/acr3)C* are the typical organization structures of *ars* operons [35], [47]. Apart from these operons, there are a few other operons derived from these main structures. In the *Burkholderiales* order, the number of operon structures was exceeded our expectation because these strains descended from a recent common ancestor. This result indicates that the *ars* operon has a high diversity of organization. Considering the recent common ancestor for these strains, multiple forms of the *ars-like* operon within *Burkholderiales* may emerge through the HGT or by gene rearrangement. In any case, this result hints at the potentially efficient movement of *ars-like* genes. However, one should keep in mind that the number of different arrangements of *ars-like* clusters may not be very accurate because some genomes are in draft status, which may split an *ars-like* cluster into more than one cluster or lead an *ars-like* cluster to separate the different genes. However, in genomic analyses, such errors occur at a very low probability. There are five main forms (>4.5%) of the *ars-like* cluster: *arsC*-*acr3*, *arsR*-*arsC*-*acr3*, *arsR*-*arsC*-*acr3*-*arsH*, *arsR*-*glo*-*arsC*-*acr3* and *arsR*-*arsC*-*acr3*-*arsC*-*arsH*. The *arsC* and *acr3* genes are shared among these five organizations, which supports a key role for these two genes in resistance to arsenic. This prediction was agreement with the opinion that *arsB/acr3* contribute to the basic resistance to arsenic in bacteria [7], [35]. Currently, several genes have been reported to be involved in the arsenic resistance system and are defined as *ars-like* genes: *arsR*, *arsA*, *arsD*,*arsB*, *acr3*,*arsC*,*arsH* [48], *arsO* [49] and *arsP* (putative membrane permease) [50]. In this study, the *glo* gene, encoding the glyoxalase/bleomycin resistance-related product, was found to be located in the *ars-like* cluster (Figure 3) in numerous *Burkholderiales* genomes. This result suggests that this gene contributes to arsenic-resistance, as functionally related genes tend to cluster together.

Arsenate-respiring bacteria reduce AsV to As III and affect the speciation and mobilization of arsenic in various locales worldwide, especially in anaerobic conditions. In these 188 genomes, the AsV respiratory reductase gene *arrAB* was only found in *P. excrementihominis* YIT 11859. This strain is a strictly anaerobic bacterium that was isolated from the human gut [51]. In *Shewanella* sp. ANA-3, expression of *arrAB* was silent under aerobic conditions, and these two genes were predicted to be obtained through HGT [36]. Therefore, the fact that *arrAB* genes were not identified in most of the 188 strains may be explained by the requirement for anaerobic conditions for AsV respiratory reductase to function [11], as most strains came from aerobic niches (Table S1).

As for the *aio-like* gene, multiple lines of evidence have demonstrated that HGT plays an important role in spreading *aio-like* genes among bacteria [45]. The *aio-like* genes identified in the *R. solanacearum* PSI07 plasmid also supported the above conclusion. In this study, numerous genomes have been found to contain arsenite oxidation and phosphate-related genes (such as the *pst* transport system and *pho* regulatory element) together. A previous study showed that the phosphate transport system (Pst) flanking the *aioAB* genes could bind phosphate selectively over arsenate (at least 10^3^-fold excess), even in arsenate-rich conditions [52], which seems to weaken the relationship between arsenic and phosphorus metabolism. However, recently, it was reported that the expression of *aioAB* was under the control of the phosphate regulators *phoBR* in *A. tumefaciens* 5A [53] *.* In addition, we found that in *Agrobacterium tumefaciens* GW4 [54], the Pst1 located near the AioAB could bind both phosphate and arsenate (Wang et al., submitted to Environmental Microbiology) which suggests significant relatedness between arsenic and phosphorus metabolism.

The arsenite S-adenosylmethyltransferase encoding gene (*arsM*) was only identified in few *Burkholderiales* genomes, which may indicate a low frequency of occurrence in the *Burkholderiales* order. The *arsM* gene was widely found in bacteria, archaea and eukarya (excluding plants) and displayed a high diversity of sequence [1]. However, a small number of ArsM are currently available in the NCBI proteins database compared with *ars-like* genes. One possible reason for the low number of *Burkholderiales* strains harboring *arsM* may be that As III biomethylation is not a primary pathway for bacterial arsenic detoxification. Bacteria have two mechanisms to deal with As III in vivo, As III biomethylation and As III oxidation. These two mechanisms share the common substrate of As III. In *Burkholderiales*, we found that the four potential As III biomethylation strains did not contain the *aioAB* genes in their genomes. However, the *arsM* gene was identified in some of the 39 arsenite-oxidizer genomes, such as *Candidatus Nitrospira* defluvii (Locus_tag, NIDE3709) and *Thiocapsa marina* 5811 (Locus_tag, ThimaDRAFT_0102), which suggests that the pathways of As III biomethylation and As III oxidation could coexist in one strain.

## Supporting Information

Figure S1 **The flowchart displaying the process used to determine the arsenic-related genes in** ***Burkholderiales*** **genomes.**
(TIF)

Figure S2 **The 16S rRNA genes based phylogenetic tree of 184** ***Burkholderiales*** **strains.** Four strains (*Acidovorax avenae* subsp. *avenae* RS-1, *Bordetella holmesii* 44057, *Burkholderia ambifaria* IOP40-10 and *Burkholderia ambifaria* MEX-5) are not involved in this phylogenetic analysis due to the 16S rRNA genes not identified in their genomes.
(DOCX)

Table S1 **The isolation sources of 188** ***Burkholderiales*** **strains obtained from literature in order to be classified in nine groups according to their original habitats.**
(XLSX)

Table S2 **The detail distribution of arsenic-related genes in 188** ***Burkholderiales*** **genomes.**
(XLSX)

Table S3 **The names of 188 strains to construct a phylogenetic tree based on 10 core genes from their genomes (** **Figure 2** **, left) and the original data shown the presence or absence of arsenic-related genes (** **Figure 2** **, right).**
(XLSX)
