# Supplementary figures and images for: Genomic Evidence Reveals the Extreme Diversity and Wide Distribution of the Arsenic-Related Genes in Burkholderiales
Source: PLoS One. 2014 Mar 14;9(3):e92236. doi: 10.1371/journal.pone.0092236 (PMC3954881; doi:10.1371/journal.pone.0092236)

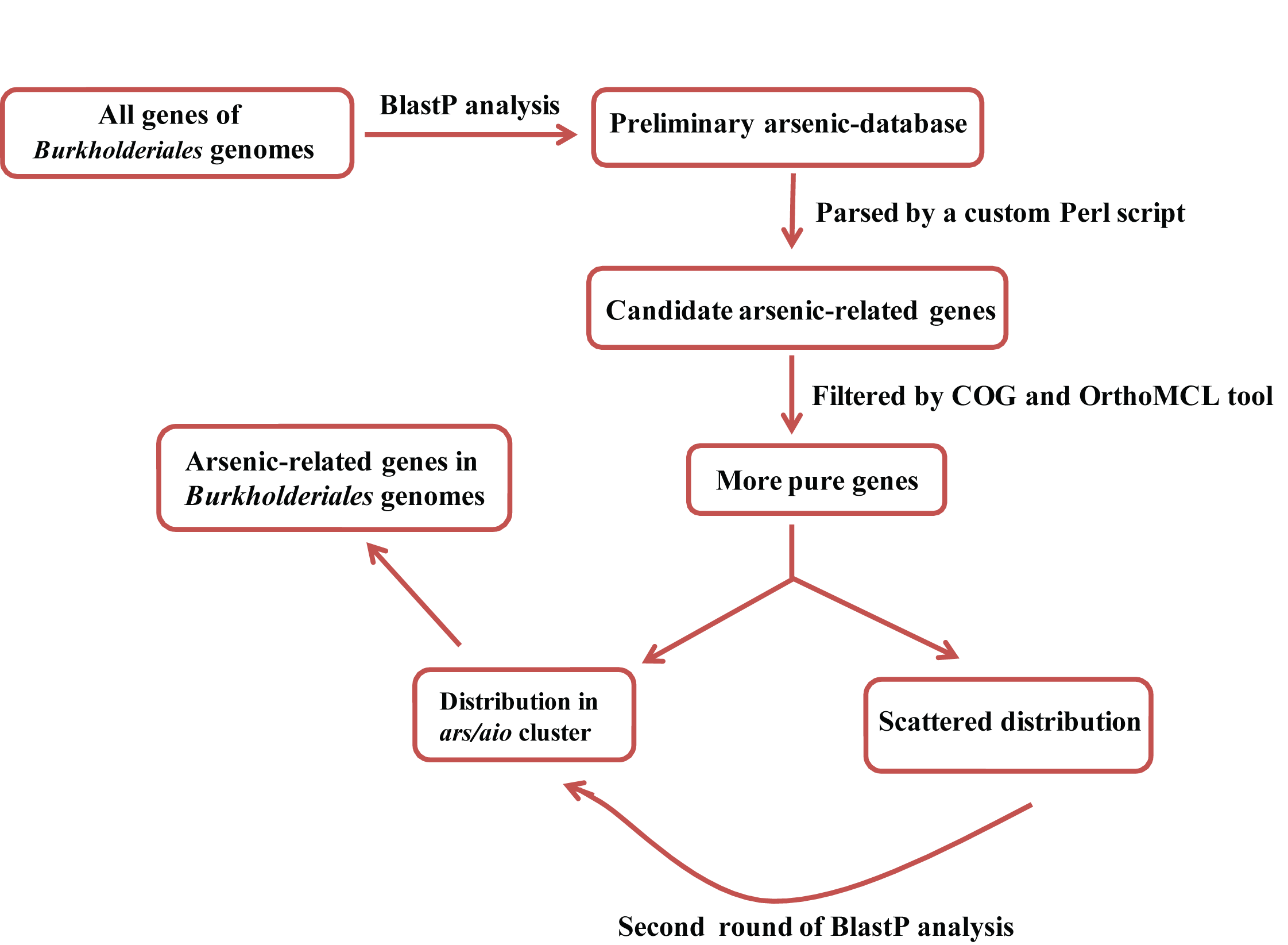

Supplement: Figure S1 — The flowchart displaying the process used to determine the arsenic-related genes in Burkholderiales genomes. (TIF) [file pone.0092236.s001.tif]
