# Supplementary material for: Genomic Evidence Reveals the Extreme Diversity and Wide Distribution of the Arsenic-Related Genes in Burkholderiales
Source: PLoS One. 2014 Mar 14;9(3):e92236. doi: 10.1371/journal.pone.0092236 (PMC3954881; doi:10.1371/journal.pone.0092236)
Supplement: Figure S2 — The 16S rRNA genes based phylogenetic tree of 184 Burkholderiales strains. Four strains (Acidovorax avenae subsp. avenae RS-1, Bordetella holmesii 44057, Burkholderia ambifaria IOP40-10 and Burkholderia ambifaria MEX-5) are not involved in this phylogenetic analysis due to the 16S rRNA genes not identified in their genomes. (DOCX) [file pone.0092236.s002.docx]

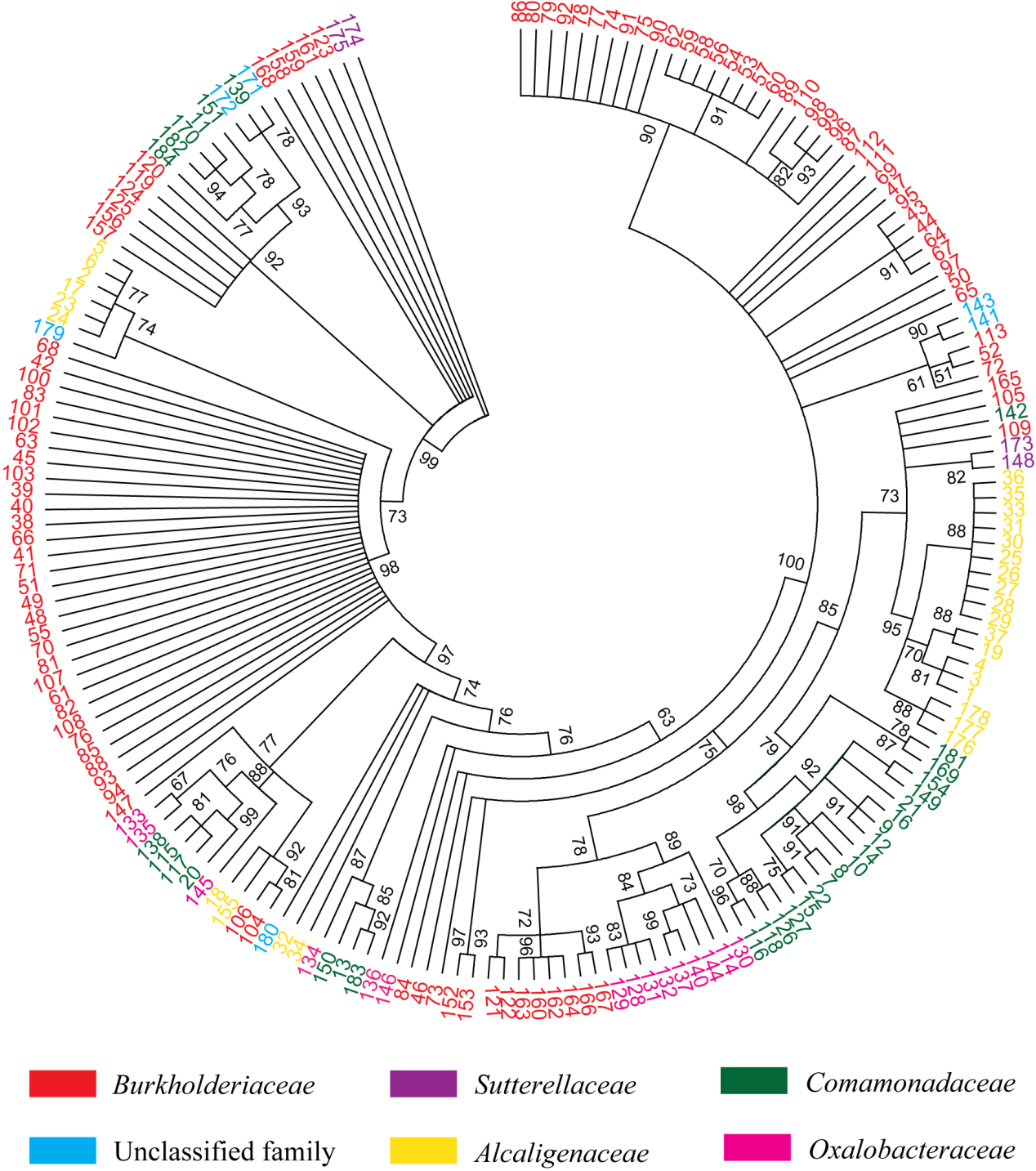


The arabic numerals indicate the strains’ names showed as following:

1 *Achromobacter arsenitoxydans* SY8

2 *Achromobacter piechaudii* ATCC 43553

3 *Achromobacter piechaudii* HLE

4 *Achromobacter xylosoxidans* A8

5 *Achromobacter xylosoxidans* AXX-A

6 *Achromobacter xylosoxidans* C54

7 *Acidovorax avenae* subsp. *avenae* ATCC 19860

8 *Acidovorax citrulli* AAC00-1

9 *Acidovorax delafieldii* 2AN

10 *Acidovorax ebreus* TPSY

11 *Acidovorax radicis* N35

12 *Acidovorax radicis* N35v

13 *Acidovorax* sp. CF316

14 *Acidovorax* sp. JS42

15 *Acidovorax* sp. KKS102

16 *Acidovorax* sp. NO-1

17 *Advenella kashmirensis* WT001

18 *Alcaligenes faecalis* subsp. *faecalis* NCIB 8687

19 *Alcaligenes* sp. HPC1271

20 *Alicycliphilus denitrificans* BC

21 *Alicycliphilus denitrificans* K601

22 *Alicycliphilus* sp. CRZ1

23 *Bordetella avium* 197N

24 *Bordetella bronchiseptica* 253

25 *Bordetella bronchiseptica* MO149

26 *Bordetella bronchiseptica* RB50

27 *Bordetella parapertussis* 12822

28 *Bordetella parapertussis* Bpp5

29 *Bordetella pertussis* 18323

30 *Bordetella pertussis* B0558

31 *Bordetella pertussis* B1193

32 *Bordetella pertussis* B1831

33 *Bordetella pertussis* B1834

34 *Bordetella pertussis* B1917

35 *Bordetella pertussis* CS

36 *Bordetella pertussis* Tohama I

37 *Bordetella petrii* DSM 12804

38 *Burkholderia ambifaria* AMMD

39 *Burkholderia ambifaria* MC40-6

40 *Burkholderia cenocepacia* AU 1054

41 *Burkholderia cenocepacia* BC7

42 *Burkholderia cenocepacia* HI2424

43 *Burkholderia cenocepacia* J2315

44 *Burkholderia cenocepacia* K56-2Valvano

45 *Burkholderia cenocepacia* MC0-3

46 *Burkholderia cenocepacia* PC184

47 *Burkholderia cepacia* GG4

48 *Burkholderia gladioli* BSR3

49 *Burkholderia glumae* AU6208

50 *Burkholderia glumae* BGR1

51 *Burkholderia glumae* LMG 2196

52 *Burkholderia graminis* C4D1M

53 *Burkholderia mallei* 2002721280

54 *Burkholderia mallei* ATCC 10399

55 *Burkholderia mallei* ATCC 23344

56 *Burkholderia mallei* FMH

57 *Burkholderia mallei* GB8 horse 4

58 *Burkholderia mallei* JHU

59 *Burkholderia mallei* NCTC 10229

60 *Burkholderia mallei* NCTC 10247

61 *Burkholderia mallei* PRL-20

62 *Burkholderia mallei* SAVP1

63 *Burkholderia multivorans* ATCC 17616

64 *Burkholderia multivorans* ATCC BAA-247

65 *Burkholderia multivorans* CF2

66 *Burkholderia multivorans* CGD1

67 *Burkholderia multivorans* CGD2

68 *Burkholderia multivorans* CGD2M

69 *Burkholderia oklahomensis* C6786

70 *Burkholderia oklahomensis* EO147

71 *Burkholderia phymatum* STM815

72 *Burkholderia phytofirmans* PsJN

73 *Burkholderia pseudomallei* 1026a

74 *Burkholderia pseudomallei* 1026b

75 *Burkholderia pseudomallei* 1106a

76 *Burkholderia pseudomallei* 1106b

77 *Burkholderia pseudomallei* 1258a

78 *Burkholderia pseudomallei* 1258b

79 *Burkholderia pseudomallei* 1655

80 *Burkholderia pseudomallei* 1710a

81 *Burkholderia pseudomallei* 1710b

82 *Burkholderia pseudomallei* 305

83 *Burkholderia pseudomallei* 354a

84 *Burkholderia pseudomallei* 354e

85 *Burkholderia pseudomallei* 406e

86 *Burkholderia pseudomallei* 576

87 *Burkholderia pseudomallei* 668

88 *Burkholderia pseudomallei* Bp22

89 *Burkholderia pseudomallei* BPC006

90 *Burkholderia pseudomallei* K96243

91 *Burkholderia pseudomallei* MSHR346

92 *Burkholderia pseudomallei* Pakistan 9

93 *Burkholderia pseudomallei* Pasteur 52237

94 *Burkholderia pseudomallei* S13

95 *Burkholderia pyrrocinia* CH-67

96 *Burkholderia rhizoxinica* HKI 454

97 *Burkholderia* sp. 383

98 *Burkholderia* sp. CCGE1001

99 *Burkholderia* sp. CCGE1002

100 *Burkholderia* sp. CCGE1003

101 *Burkholderia* sp. Ch1-1

102 *Burkholderia* sp. H160

103 *Burkholderia* sp. KJ006

104 *Burkholderia* sp. SJ98

105 *Burkholderia* sp. TJI49

106 *Burkholderia* sp. YI23

107 *Burkholderia terrae* BS001

108 *Burkholderia thailandensis* Bt4

109 *Burkholderia thailandensis* E264

110 *Burkholderia thailandensis* MSMB43

111 *Burkholderia thailandensis* TXDOH

112 *Burkholderia vietnamiensis* G4

113 *Burkholderia xenovorans* LB400

114 *Collimonas fungivorans* Ter331

115 *Comamonas testosteroni* ATCC 11996

116 *Comamonas testosteroni* CNB-2

117 *Comamonas testosteroni* KF-1

118 *Comamonas testosteroni* S44

119 *Cupriavidus basilensis* OR16

120 *Cupriavidus metallidurans* CH34

121 *Cupriavidus necator* HPC(L)

122 *Cupriavidus necator* N-1

123 *Cupriavidus* sp. BIS7

124 *Cupriavidus* sp. HMR-1

125 *Cupriavidus taiwanensis* LMG 19424

126 *Delftia acidovorans* SPH-1

127 *Delftia* sp. Cs1-4

128 *Herbaspirillum frisingense* GSF30

129 *Herbaspirillum huttiense* subsp. *putei* IAM15032

130 *Herbaspirillum lusitanum* P6-12

131 *Herbaspirillum seropedicae* Os34

132 *Herbaspirillum seropedicae* Os45

133 *Herbaspirillum seropedicae* SmR1

134 *Herbaspirillum* sp. CF444

135 *Herbaspirillum* sp. GW103

136 *Herbaspirillum* sp. YR522

137 *Herminiimonas arsenicoxydans*

138 *Hydrogenophaga* sp. PBC

139 *Hylemonella gracilis* ATCC 19624

140 *Janthinobacterium* sp. PAMC 25724

141 *Leptothrix cholodnii* SP-6

142 *Limnohabitans* sp. Rim47

143 *Methylibium petroleiphilum* PM1

144 *Oxalobacter formigenes* HOxBLS

145 *Oxalobacter formigenes* OXCC13

146 *Oxalobacteraceae* bacterium IMCC9480

147 *Pandoraea* sp. B-6

148 *Parasutterella excrementihominis* YIT 11859

149 *Polaromonas naphthalenivorans* CJ2

150 *Polaromonas* sp. CF318

151 *Polaromonas* sp. JS666

152 *Polynucleobacter necessarius* subsp. *asymbioticus* QLW-P1DMWA-1

153 *Polynucleobacter necessarius* subsp. *necessarius* STIR1

154 *Pseudacidovorax intermedius* NH-1

155 *Pusillimonas noertemannii* BS8

156 *Ralstonia eutropha* H16

157 *Ralstonia eutropha* JMP134

158 *Ralstonia pickettii* 12D

159 *Ralstonia pickettii* 12J

160 *Ralstonia solanacearum* CFBP2957

161 *Ralstonia solanacearum* GMI1000

162 *Ralstonia solanacearum* Po82

163 *Ralstonia solanacearum* PSI07

164 *Ralstonia solanacearum* UW551

165 *Ralstonia solanacearum* Y45

166 *Ralstonia* sp. 5 2 56FAA

167 *Ralstonia* sp. 5 7 47FAA

168 *Ralstonia* sp. PBA

169 *Ramlibacter tataouinensis* TTB310

170 *Rhodoferax ferrireducens* T118

171 *Rubrivivax benzoatilyticus* JA2

172 *Rubrivivax gelatinosus* IL144

173 *Sutterella parvirubra* YIT 11816

174 *Sutterella wadsworthensis* 2 1 59BFAA

175 *Sutterella wadsworthensis* 3 1 45B

176 *Taylorella asinigenitalis* MCE3

177 *Taylorella equigenitalis* ATCC 35865

178 *Taylorella equigenitalis* MCE9

179 *Thiomonas intermedia* K12

180 *Thiomonas* sp. 3As

181 *Variovorax paradoxus* EPS

182 *Variovorax paradoxus* S110

183 *Variovorax* sp. CF313

184 *Verminephrobacter eiseniae* EF01-2
